# Predicting pathological complete response after neoadjuvant chemotherapy: A nomogram combining clinical features and ultrasound semantics in patients with invasive breast cancer

**DOI:** 10.3389/fonc.2023.1117538

**Published:** 2023-03-22

**Authors:** Ke-Nie Wang, Ya-Jiao Meng, Yue Yu, Wen-Run Cai, Xin Wang, Xu-Chen Cao, Jie Ge

**Affiliations:** ^1^ The First Department of Breast Cancer, Tianjin Medical University Cancer Institute and Hospital, National Clinical Research Center for Cancer, Tianjin, China; ^2^ Key Laboratory of Cancer Prevention and Therapy, Tianjin, China; ^3^ Tianjin’s Clinical Research Center for Cancer, Tianjin, China; ^4^ Key Laboratory of Breast Cancer Prevention and Therapy, Tianjin Medical University, Ministry of Education, Tianjin, China; ^5^ Department of Obstetrics & Gynecology, Tianjin 4th Centre Hospital, Tianjin, China

**Keywords:** diagnostic imaging, invasive breast cancer, nomogram, treatment outcome, cutoffs

## Abstract

**Background:**

Early identification of response to neoadjuvant chemotherapy (NAC) is instrumental in predicting patients prognosis. However, since a fixed criterion with high accuracy cannot be generalized to molecular subtypes, our study first aimed to redefine grades of clinical response to NAC in invasive breast cancer patients (IBC). And then developed a prognostic model based on clinical features and ultrasound semantics.

**Methods:**

A total of 480 IBC patients were enrolled who underwent anthracycline and taxane-based NAC between 2018 and 2020. The decrease rate of the largest diameter was calculated by ultrasound after NAC and their cut-off points were determined among subtypes. Thereafter, a nomogram was constructed based on clinicopathological and ultrasound-related data, and validated using the calibration curve, receiver operating characteristic (ROC) curve, decision curve analysis (DCA), and clinical impact curve (CIC).

**Results:**

The optimal cut-off points for predicting pCR were 53.23%, 51.56%, 41.89%, and 53.52% in luminal B-like (HER2 negative), luminal B-like (HER2 positive), HER2 positive, and triple-negative, respectively. In addition, time interval, tumor size, molecular subtypes, largest diameter decrease rate, and change of blood perfusion were significantly associated with pCR (all *p* < 0.05). The prediction model based on the above variables has great predictive power and clinical value.

**Conclusion:**

Taken together, our data demonstrated that calculated cut-off points of tumor reduction rates could be reliable in predicting pathological response to NAC and developed nomogram predicting prognosis would help tailor systematic regimens with high precision.

## Introduction

Breast cancer is the most common newly diagnosed malignancy, accounted for over 30% among female (1). Neoadjuvant chemotherapy (NAC) was the first-line regimen for the locally advanced with equivalent efficacy to adjuvant chemotherapy, striving for fostering possibility of receiving surgery therapy and reducing tumor burden (2). The indications for NAC have now been extended to include high-risk patients, as well as those pursuing breast-conservation treatment and eligibility for sentinel lymph node biopsy as an alternative to axillary lymph node dissection upon fulfillment of specific requirements (3–6). The administration of NAC prior to surgery therapy attenuates cell proliferation and vascularity; meanwhile, it serves as a visualization window for the efficacy of chemotherapeutics (7, 8). As a characteristic of breast cancer with high heterogeneity, differentiated manifestations are represented between individuals and molecular subtypes following NAC. The ideal treatment outcome is a pathological complete response (pCR), which has become a decision node in systematic therapy. Patients achieved pCR have much improved outcomes compared to those with residual cancer burden, which prolongs survival time and decrease risk of distant metastasis (9, 10). The strongest association have been illustrated between prolonged long-term survival and high-risk patients with the most aggressive clinical characteristics of triple-negative or HER2 positive breast cancer (3, 9–11). For patients with non-pCR following NAC, subsequent utility of adjuvant intensive treatment still have access to reduce relapse rates after surgery, such as capecitabine in triple-negative (12, 13), T-DM1 in HER2 positive which has been approved by clinical practice guidelines worldwide (14). However, unavoidable side effects exist with classically effective treatment regimens based on anthracyclines and taxanes, which have been proven to increase non-breast-cancer death in previous researches, along with the marked benefit of reduced mortality or recurrence rates (4). Few events with anthracycline and paclitaxel-based chemotherapy regimens increase the risk of leukemia, with anthracyclines additionally increasing the risk of heart disease where patients exhibit propensity to irreversible decrease in ejection fraction (4). Thus, accurate judgments about the response to NAC are required to prevent excessive drug-related toxicity and adjust regimens in a timely manner.

Evaluation of clinical response has widely relied on the Response Evaluation Criteria in Solid Tumors (RECIST) 1.1 criteria which identified the largest diameter as the most common parameter, but did not recommend breast ultrasound as the assessment tool (15). However, ultrasound imaging remains the most popular tool for patients due to its low cost, lack of facility limitations and convenience. Importantly, ultrasound has been shown to be accurate, especially in the assessment of tumor size which is the most important indicator for evaluating response (16–18). Many previous studies have demonstrated only minor differences in tumor size between ultrasound and pathological measurement, which are attributed to unclear margins, edge perception and histological features (19, 20). Therefore, change in the largest diameter measured by breast ultrasound is one of the most commonly used clinical formats to balance generalizability and accuracy when assessing clinical response to NAC. According to RECIST 1.1 criteria, a 30% decline or more is for determining a partial responder following NAC, while a 20% increase or greater is for a progressive disease. However, with the exception of pCR, response grades are ambiguous since the mismatches in terms of clinical grading criteria and pathological classifications regardless of molecular subtype. The AUC value was only about 0.6 *via* ultrasound assessment when comparing tumor decrease less than 30% with pathological poor response with the definition of grade 1/2 in the Miller-Payne classification (21). To date, a few studies have attempted to determine the clinical cut-off points for predicting pathological response. The rate of reduction was set at 23% of tumor size to predict pCR after two cycles, but the differences among molecular subtypes were not defined (22). The optimal values for predicting responses among various molecular subtypes remain uncertain. Our study sought to set up the exact cutoff values in molecular subtypes and build a universal model using generous instruments based on existing cases, which possesses great potential of clinical benefits in therapeutic settings.

Here, we first calculated the optimal clinical cut-off points for the largest diameter decrease that would predict different grades of pathological response among renewable molecular subtypes. And then, combined with other clinicopathological variables and parameters of breast ultrasound, we evaluated variables to predict pCR status and developed a prognostic nomogram, which would assist in decision-making processes.

## Materials and methods

### Patient population

We enrolled patients with pathologically confirmed invasive breast cancer (IBC) who underwent at least 4 cycles of NAC with anthracycline (i.e., Doxorubicin, Epirubicin, and Pirarubicin) and taxane (i.e., Docetaxel, Paclitaxel, and Taxol) for every 3 weeks. The patients were required to undergo clinical evaluations with ultrasound breast exams before and after NAC. After the last course, either mastectomy or breast conservation plus axillary lymph node dissection was then performed within 4 weeks. Patients with bilateral breast cancer, distant metastasis, suspected relapse or recurrence, other additional cancers, as well as those with inoperable tumors were excluded from the study. Male patients and those with incomplete data on medical and imaging records were excluded. In addition, patients whose preoperative biopsy lacked immunohistochemistry (IHC) or whose HER2 status were 2+ without fluorescence *in situ* hybridization (FISH) analysis were also excluded from the analysis.

Between January 2018 and December 2020, female patients who met the inclusion criteria at the Tianjin Medical University Cancer Institute and Hospital (Tianjin, China) were documented. A total of 480 patients were enrolled in this analysis, and they were randomly allocated into the training and validation cohorts at a ratio of 7:3. Diagnostic age was extracted from the general information and menopausal status was extracted from their personal history, which consisted of premenopausal and postmenopausal features. The time interval was defined from the presence of the first symptom to the start of treatment. This study was approved by the Institutional Review Board of Tianjin Medical University (Number: bc2022211).

### Imaging and histopathology data

Ultrasound examination was performed before the first cycle and after the final cycle of NAC. The patients were assessed by certified radiologists using the LOGIQ E9 (General Electric Co., USA) ultrasound machine with a 6–15 MHz linear transducer. Data on the largest diameter and blood perfusion were extracted from the patient reports. The largest diameter was categorized following the clinical T stage, and then the percentage of the largest diameter decrease was calculated after NAC. Based on the reports, blood perfusion of the tumor was categorized as scarce or abundant and then compared at the end of the course using a color Doppler ultrasound vascular pattern.

Prior to NAC, ultrasound-guided biopsies of the primary tumor were performed with a 14-gauge needle, and specimens were stained and then microscopically assessed in the department of Pathology. Besides, we evaluated ER, PR, HER2 and Ki-67 status using IHC. ER and PR were positive with more than 1% staining of the cancer cells. HER2 was considered positive with a staining intensity score of 3+ and considered negative with a score of 0 or 1+. Furthermore, FISH analysis was used to determine HER2 status in tumors with a score of 2+. 20% expression of Ki-67 was used to distinguish a high proliferation index. The patients were then stratified into four molecular subtypes according to the 2017 St. Gallen Consensus meeting and included luminal A-like, luminal B-like, HER2 positive (non-luminal) and triple-negative subtypes. The luminal A-like subtype was defined as ER positive, with PR of more than 20%, HER2 negative and with a low Ki-67 proliferation index. On the other hand, luminal B-like group was defined as ER positive with either PR expression of less than 20% or a high Ki-67 proliferation index, which was further divided into HER2 negative and HER2 positive subgroups. HER2 positive (non-luminal) was defined as HER2 positive with any Ki-67 expression and triple-negative as ER negative, PR negative, and HER2 negative. Further, Tumors with greater than 10% stained cells were considered p53 positive. Those with more than 1% stained cells were considered as AR positive.

After the last course and surgery, pathological assessment of surgical specimens was performed, and then the pathological response was analyzed based on the criteria outlined by the Japanese Breast Cancer Society, which was considered the reference standard (23, 24). Grade 3 was considered the absence of any invasive cancer cells in the primary tumor, which was equivalent to the definition of pCR. Grade 2 encompassed tumors with more than 2/3 cell changes or those that have almost achieved pCR with few remaining invasive tumor cells. Grade 1 referred to cancer cells with slight changes or fewer than 2/3 of tumor cells with significant changes. Grade 0 had virtually no changes in tumor cells. This study did not consider nodal status.

### Data analysis

The primary aim of this study was to define the optimal cut-off in changes of the largest diameter of ultrasound assessment that would predict pCR and grades of pathological response to NAC among the molecular subtypes. Receiver operating characteristic (ROC) curve analyses and the area under the curve (AUC) with 95% confidence intervals (CIs) were carried out to identify cut-off points of the largest diameter decrease using the Youden method for different response grades in the training cohort. Accuracy, sensitivity, specificity, diagnostic odds ratio (DOR), false positive rate (FPR), positive predictive value (PPV), negative predictive value (NPV), and Youden index for each cut-off value were systematically calculated. The secondary aim of the study was to develop a multivariable logistic regression model to evaluate the prognostic usefulness of the predictors in patients who achieved pCR following NAC. The variables, together with the largest diameter decrease stratified by the calculated cut-off point, were incorporated into the multivariable logistic regression model if *p* < 0.1 in the univariable analysis within the training cohort. As ER, PR, HER2, and Ki-67 status were used to stratify various molecular subtypes, they were not included in the univariable analysis. Independent predictors were significantly identified with a *p* < 0.05 in the multivariable logistic regression model, which were used to establish a nomogram to predict the outcome of patients who achieved pCR. The calibration curve, concordance-index (C-index), ROC, decision curve analysis (DCA), and clinical impact curve (CIC) were employed to validate the calibration, discrimination, and clinical usefulness of the developed prognostic model.

### Statistical analysis

All statistical analyses were performed in SPSS version 22.0 (IBM Corporation, Armonk, NY, USA) and R software version 4.0.5 (https://www.r-project.org) using R packages such as rms, readr, foreign, pROC, car, rmda, and ggplot2. The continuous variable with non-normal distribution was represented as the median and quartiles, while categorical variables were represented as frequency and percentage. Differences between the training and the validation group were compared using the Mann-Whitney test among continuous variable and chi-square tests among categorical variables. The ROC, AUC, and 95% CIs were calculated by R software. In addition, univariable and multivariable logistic regression analyses were conducted using SPSS software. The development and validation of the nomogram was performed by the R software. All *p* values were calculated as two-sided and significance was set at *p* < 0.05.

## Results

### Patient cohort and characteristics

Between January 2018 and December 2020, a total of 480 women who underwent breast ultrasound examination before and after a completed course of NAC were included in this study, following the previously described patient selection criteria (**Supplementary Figure 1**). We analyzed the baseline characteristics of the patients in the training and validation cohorts as shown in **Table 1**. The analysis showed that the median age for the patients was 49 years. 56.9% of patients (n = 273) were in premenopausal status while 43.1% (n = 207) were in the postmenopausal phase. 354 patients underwent treatment within 3 months after the first symptom was noticed, while 126 patients underwent treatment 3 months later. Tumor size in most of the patients was between 2 cm and 5 cm (n = 332, 69.2%), followed by larger than 5 cm (n = 98, 20.4%). Analysis of the newly defined molecular subtypes showed that 45 patients (9.4%) were luminal A-like; 264 patients (55.0%) were luminal B-like (HER2 negative); 63 patients (13.1%) were luminal B-like (HER2 positive); 37 patients (7.7%) were HER2 positive (non-luminal) while the remaining 71 patients (14.8%) were triple-negative. There were 174 (36.3%) and 403 (84.0%) patients who were considered positive for p53 and AR status, respectively.

**Table 1:** Baseline characteristics of patients with invasive breast cancer.

Variables	All Patients (n=480)		Training cohort(n=336)		Validation cohort (n=144)		*P* Value
	n	%	n	%	n	%	
**Age at diagnosis** **years, median (range)**	49.0 (41.3-57.0)		49.5 (41.0-57.0)		48 (42.0-57.8)		0.664
**Menopausal status**							0.410
Premenopausal	273	56.9	187	55.7	86	59.7	
Postmenopausal	207	43.1	149	44.3	58	40.3	
**Time interval, months**							0.856
<= 3	354	73.8	247	73.5	107	74.3	
> 3	126	26.2	89	26.5	37	25.7	
**Tumor size, cm**							0.506
<= 2	50	10.4	37	11.0	13	9.0	
> 2 and <= 5	332	69.2	227	67.6	105	72.9	
> 5	98	20.4	72	21.4	26	18.1	
**ER status**							0.127
Negative	108	22.5	82	24.4	26	18.1	
Positive	372	77.5	254	75.6	118	81.9	
**PR status**							0.309
Negative	173	36.0	126	37.5	47	32.6	
Positive	307	64.0	210	62.5	97	67.4	
**HER2 status**							0.624
Negative	100	20.8	264	78.6	116	80.6	
Positive	380	79.2	72	21.4	28	19.4	
**Ki-67 status**							0.191
Low (< 20%)	62	12.9	39	11.6	23	16.0	
High (>= 20%)	418	87.1	297	88.4	121	84.0	
**Molecular subtypes**							0.634
Luminal A-like	45	9.4	30	8.9	15	10.4	
Luminal B-like (HER2 negative)	264	55.0	181	53.9	83	57.6	
Luminal B-like (HER2 positive)	63	13.1	43	12.8	20	13.9	
HER2 positive (non-luminal)	37	7.7	29	8.6	8	5.6	
Triple-negative	71	14.8	53	15.8	18	12.5	
**p53 status**							0.431
Negative	306	63.7	218	64.9	88	61.1	
Positive	174	36.3	118	35.1	56	38.9	
**AR status**							0.098
Negative	77	16.0	60	17.9	17	11.8	
Positive	403	84.0	276	82.1	127	88.2	
**Blood perfusion**							0.663
Scarce	104	21.7	71	21.1	33	22.9	
Abundant	376	78.3	265	78.9	111	77.1	
**Change of blood perfusion**							0.662
Stable	292	60.8	200	59.5	92	63.9	
Less	169	35.2	122	36.3	47	32.6	
More	19	4	14	4.2	5	3.5	

Overall, 20.6% of the patients achieved pCR (n = 99). HER2 positive (non-luminal) had the highest pCR rate (51.4%) among the molecular subtypes, followed by triple-negative (39.4%), luminal B-like (HER2 positive; 30.2%), and luminal B-like (HER2 negative; 12.5%) (**Supplementary Table 1**). However, there was no pCR in patients with luminal A-like.

### Evaluation accuracy of cut-off values in the largest diameter decrease by breast ultrasound

The optimal cut-off for predicting pCR in the training cohort was set at 53.23%, with an accuracy of 0.774, a specificity of 0.822, and a sensitivity of 0.582 (**Supplementary Tables 2, 3**). Besides, the optimal cut-off point had a NPV of 0.888, a PPV of 0.448, a Youden index of 0.404, and AUC of 0.696 (95% CI, 0.614-0.778). Among the molecular subtypes, the cut-off values for pCR were 53.23%, 51.56%, 41.89%, and 53.52% for luminal B-like (HER2 negative), luminal B-like (HER2 positive), HER2 positive (non-luminal) and triple-negative subtypes, respectively (**Supplementary Table 2**). As for grade 2, the optimal value was set at 44.75% for all the training patients. The accuracy, specificity, sensitivity, NPV, PPV, Youden index, and AUC of the grade 2 optimal value were 0.679, 0.788, 0.554, 0.668, 0.696, 0.342, and 0.697, respectively (**Supplementary Table 4**). For grade 1, the final optimal value was set at 23.21%, with an accuracy of 0.980, specificity of 0.773, sensitivity of 1.000, NPV of 1.000, PPV of 0.979, Youden index of 0.773, and AUC of 0.765 (**Supplementary Table 5**).

### Evaluation of the value of the predictors

The calculated optimal cutoff value was rounded up to 53%, and univariate logistic regression analysis was used to analyze the association with pCR, along with other variables. The univariable logistic analysis showed that time interval, tumor size, molecular subtypes, AR status, the largest diameter decrease and change in blood perfusion were significantly associated with pCR (*p* < 0.1) (**Table 2**). These parameters were then selected for multivariable analyses. Finally, there were five independent pCR predictors, where molecular subtype emerged as the strongest predictor. HER2 positive (non-luminal) (odds ratio [OR] = 13.111; 95% CI, 4.535-37.905; *p* < 0.001), more than 53% reduction in the largest diameter (OR = 7.027; 95% CI, 3.386-14.583; *p* < 0.001) and less blood perfusion (OR = 2.549; 95% CI, 1.274-5.097; *p* = 0.008) were favorably associated with pCR. In contrast, long time interval (OR = 0.274; 95% CI, 0.108-0.697; *p* = 0.007), and tumor size greater than 2 cm (≤ 5 cm [OR = 0.162; 95% CI, 0.061-0.431; *p* < 0.001]; > 5 cm [OR = 0.226; 95% CI, 0.074-0.695; *p* = 0.009]) were inversely associated with pCR.

**Table 2:** Univariate and multivariable logistic regression model for predicting pathological complete response in the training cohort.

Variables	Univariate		Multivariable	
	OR (95% CI)	*P* value	OR (95% CI)	*P* value
**Age at diagnosis,**	0.995 (0.969-1.021)	0.683		
**Menopausal status**		0.937		
Premenopausal	Ref			
Postmenopausal	1.022 (0.597-1.751)			
**Time interval, months**		0.004		0.007
<= 3	Ref		Ref	
> 3	0.315 (0.144-0.689)		0.274 (0.108-0.697)	
**Tumor size, cm**		0.046		0.001
<= 2	Ref		Ref	
> 2 and <= 5	0.383 (0.179-0.817)		0.162 (0.061-0.431)	< 0.001
> 5	0.486 (0.201-1.174)		0.226 (0.074-0.695)	0.009
**Molecular subtypes**		< 0.001		< 0.001
Luminal A-like	–		–	
Luminal B-like (HER2 negative)	Ref		Ref	
Luminal B-like (HER2 positive)	3.695 (1.650-8.271)		3.847 (1.491-9.925)	0.005
HER2 positive (non-luminal)	9.135 (3.828-21.799)		13.111 (4.535-37.905)	< 0.001
Triple-negative	5.167 (2.488-10.734)		4.263 (1.534-11.842)	0.005
**p53 status**		0.119		
Negative	Ref			
Positive	1.543 (0.894-2.664)			
**AR status**		0.014		0.317
Negative	Ref		Ref	
Positive	0.454 (0.243-0.85)		0.611 (0.233-1.606)	
**The largest diameter decrease rate**		< 0.001		<0.001
<= 53%	Ref		Ref	
> 53%	5.812 (3.28-10.299)		7.027 (3.386-14.583)	
**Blood perfusion**		0.471		
Scarce	Ref			
Abundant	1.288 (0.647-2.562)			
**Change of blood perfusion**		< 0.001		0.029
Stable	Ref		Ref	
Less	3.850 (2.184-6.787)		2.549 (1.274-5.097)	0.008
More	0.564 (0.071-4.507)		1.158 (0.122-11.026)	0.898

### Construction and validation of a prediction nomogram

**Figure 1:**
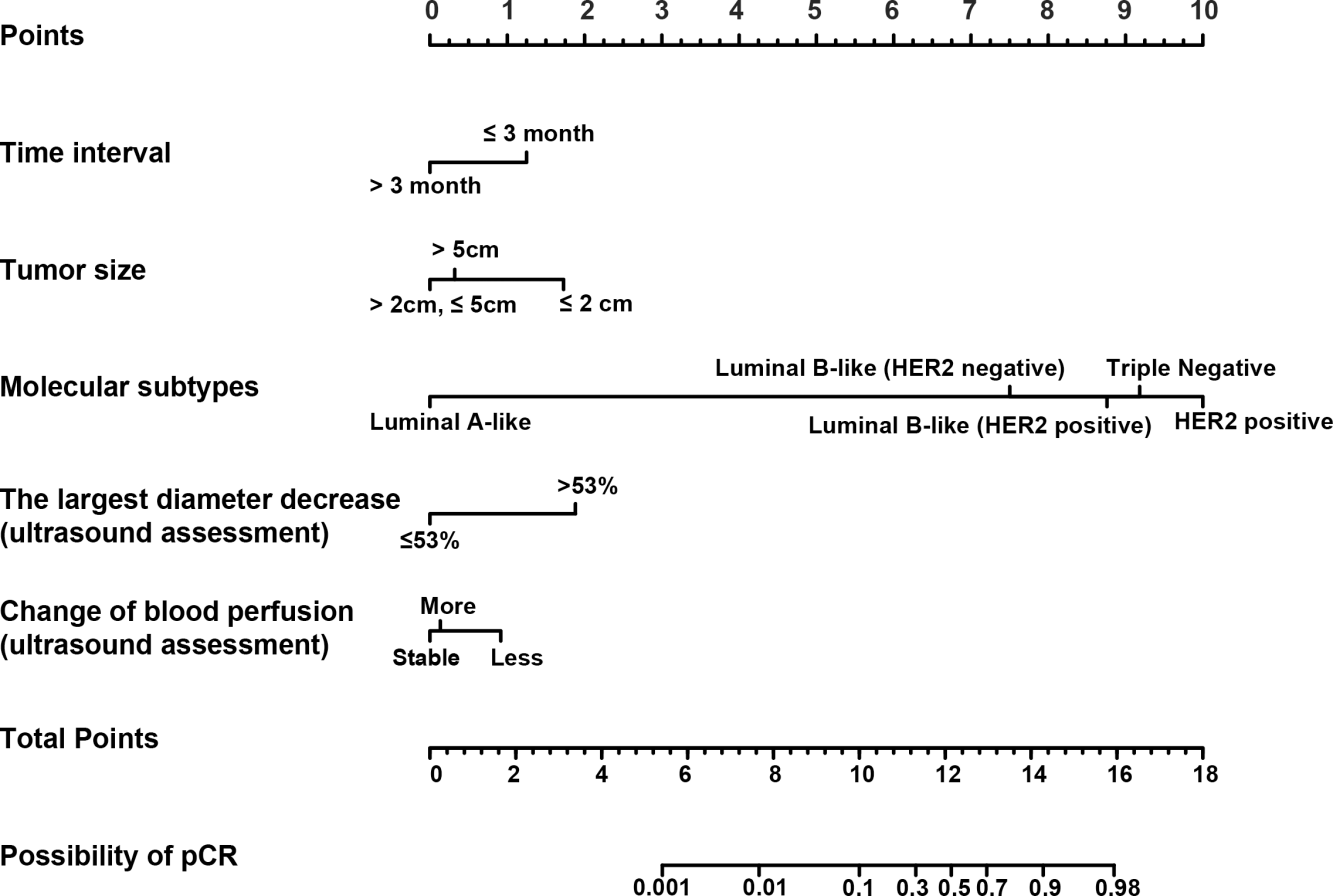
The nomogram for predicting pathological complete response (pCR) in patients with breast cancer who undergo neoadjuvant chemotherapy (NAC). Each variable was assigned a score based on its contribution to the outcome. A vertical line through each variable locates the axis that determines respective prognostic score. The total score provides an estimated probability of achieving pCR.

**Figure 2:**
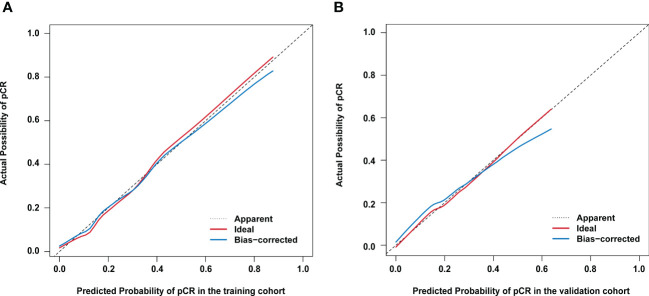
Calibration curves for actual versus predicted proportion of pathological complete response (pCR) using the nomogram **(A)** in the training cohort; and **(B)** in the validation cohort. The diagonal line represents performance of an ideal nomogram.

**Figure 3:**
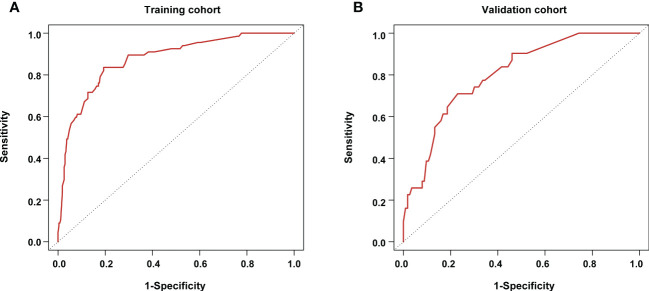
Receiving operator characteristic curves for the pathological complete response prediction model **(A)** in the training cohort; and **(B)** in the validation cohort.

**Figure 4:**
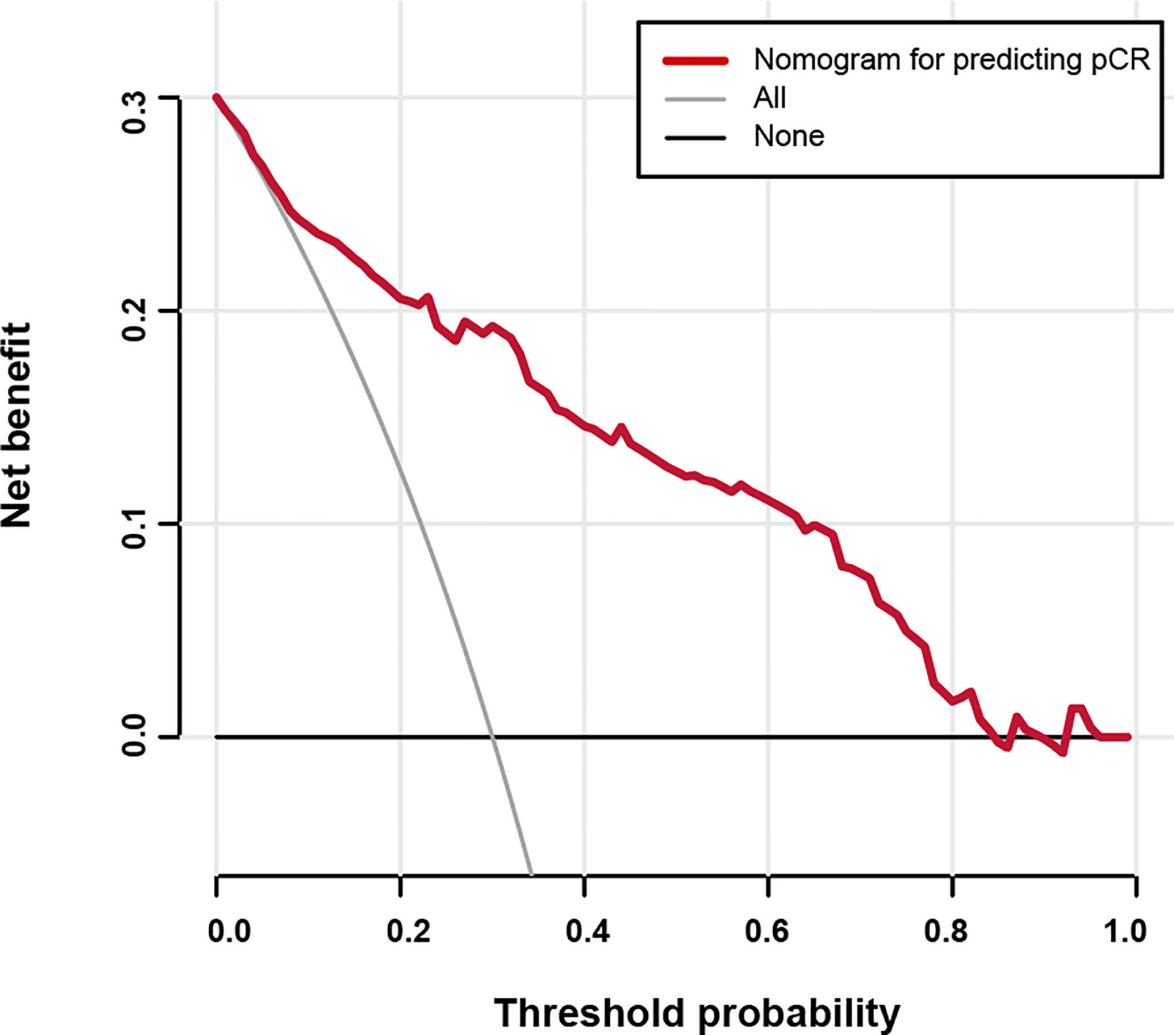
Decision curve analysis for the nomogram predicting the possibility of pathological complete response.

**Figure 5:**
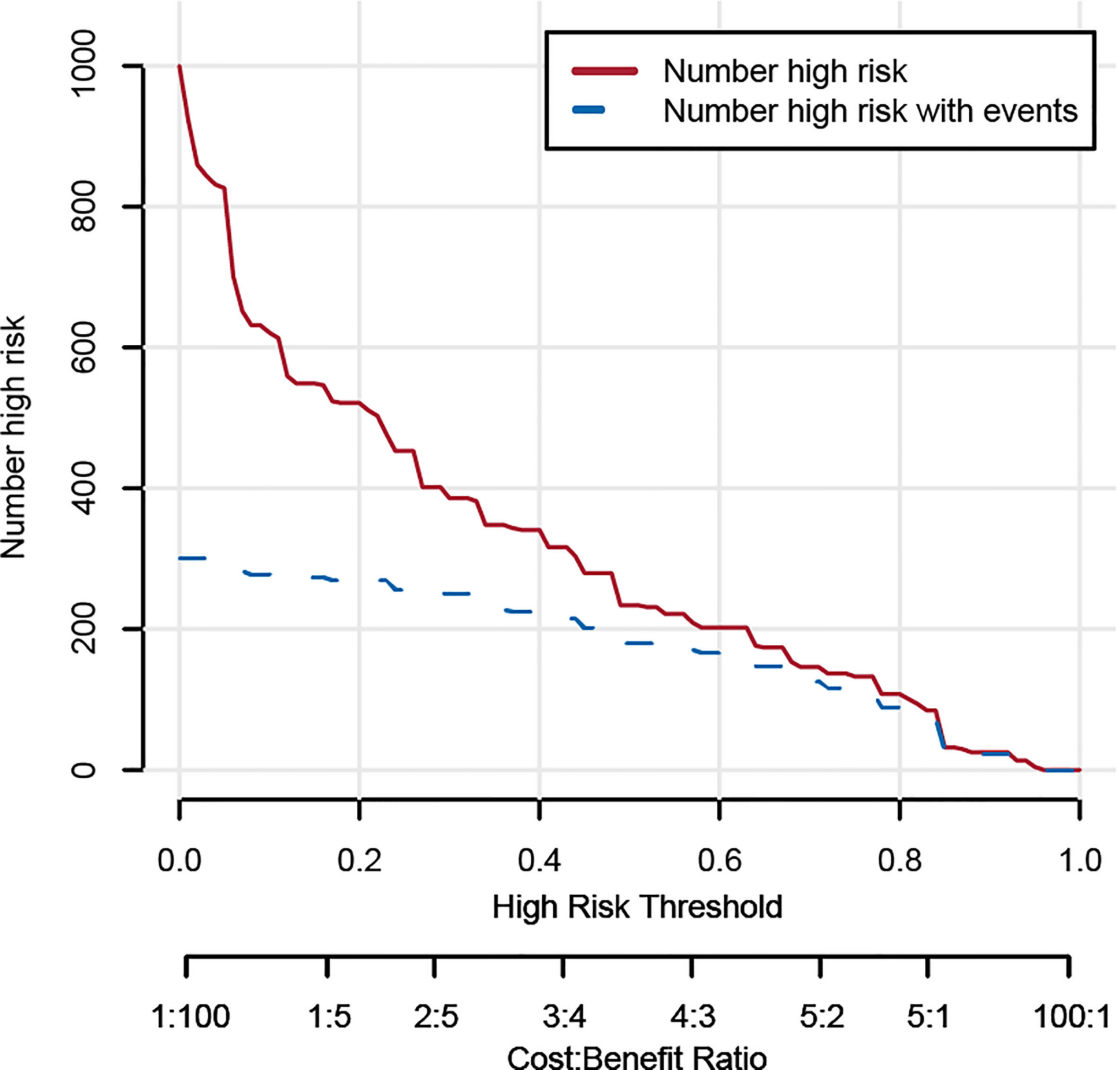
Clinical impact curve of the developed nomogram model.

Based on the results from the multivariable analysis, we constructed a nomogram (**Figure 1**) and scored each category of five variables (**Supplementary Table 6**). Calibration curves showed the nomogram had excellent consistency with actual clinical outcomes among both the training and validation cohorts (**Figure 2**). ROC was plotted with an AUC of 0.874 (95% CI, 0.827–0.921), indicating less discrimination between the model-predicted pCR and the actual outcome, and the validated AUC using the validation cohort was 0.801 (95% CI, 0.720–0.883) (**Figure 3**). Besides, the remarkable predictive performance and clinical value of the nomogram were further demonstrated by DCA and CIC. The DCA showed that the predictive model was available in a wide range (1%–85%) (**Figure 4**), which would give a net clinical benefit to a vast majority of the patients. In addition, the CIC of the training cohort revealed sufficient clinical evaluation for patients with a high probability of achieving pCR (**Figure 5**).

## Discussion

In our study, patients with aggressive subtypes such as HER2 positive and triple-negative patients had the highest pCR rates, and the results were similar to previous studies where the pCR rate in the luminal B-like subtype was less than one-fifth (25). Moreover, the achievement of pCR in these patients with aggressive subtypes was proven to be the most strongly connected to prognosis (26). We then separately calculated the optimal cut-off points in updated molecular subtypes based on the largest diameter decrease rate measured by breast ultrasound, which is for predicting pathological response grades according to different molecular subtypes. And triple-negative was highlighted as the most accurate subtype by imaging following NAC. Five independent prognostic predictors of patient achievement of pCR were determined, including time interval, tumor size, molecular subtypes, the largest diameter decrease rate, and change in blood perfusion. And the reliable clinical prognostic nomogram was established combined with clinical characteristics and breast ultrasound semantics, showing acceptable agreement, discrimination, and good clinical usefulness.

Graeser M et al. compared pCR with clinical responders by ultrasound in a low AUC ranging from 60.4% to 63.3%. However, the limited AUC values might be due to discordant clinical and pathological concepts where the study broadly defined clinical responders as clinical complete response (cCR) and partial response (cPR) (27). In contrast, comparing cPR and cCR separately for residual tumor and pCR showed great increased precision of ultrasound (28). Accurately clarified concepts on clinical and pathological correspondence, Wang et al. obtained an AUC value of up to 0.89 and a sensitivity of up to 88.1% for ultrasound monitoring with pCR (21). Besides, rather than adopting a set 30% or 100% cut-off value, precise identification of cut-off values is critical in identifying patients with various response grades among molecular subtypes. Recent studies demonstrated that the cut-off point for the reduction rate in pCR was set at 27.1% after two cycles for patients underwent NAC with an AUC greater than 0.82 (21). We investigated the most reliable points in predicting different pathological response, in relation to the molecular subtypes rather than the unitary standard. The pCR cut-off point was about 53% among the cured IBC patients, with a range of 41.89% to 53.52% among the different molecular subtypes. Triple-negative tumors had the best accuracy (0.925), while luminal tumors had the worst.

The assessed effectiveness of breast ultrasound to identify pCR with its cut-offs was associated with high sensitivity and DOR, a parameter that combines sensitivity and specificity, while reliable monitoring of non-response was required for low FPR (29). Our data showed that triple-negative phenotype demonstrated ability to predict pCR with its cut-off point in the highest accuracy, which might be explained by the growth pattern with a limited border markedly distinguished from the surrounding (30). However, luminal tumor yielded poor predictive performance with low sensitivity. This data was in sync with a previous study which showed that the assessment accuracy was significantly dependent on molecular subtypes and the lowest accuracy was found in luminal A and B subtypes with independent prediction of hormone receptor status positive (31). The poor prediction might be because of interfacial growth pattern that tumor cells invade along the duct and nourishment by surrounding angiogenesis (30).

Additionally, primary tumors were invaded by fibrous tissue due to cancer cells hypoxia and form fragment, causing no significant change in the images under ultrasound measurement (8). Therefore, there is need for further imaging approaches to monitor response in luminal subtypes. Our results showed that, with the exception of luminal A-like diseases, FPR achieved 0% for each subtype at their respective cut-off points in distinguishing non-responders (grade 1) from the entire cohort, indicating quite reliability for monitoring patients with no response. Thus, failure of the largest diameter decrease to reach cut-off points among molecular subtypes was a signal for change of regimens or reconsideration of operation time. For HER2 positive, the 0% cut-off value might because almost the patients responded with different rates of the largest diameter decrease, which might have been a limitation of the sample size.

As demonstrated by our data, time interval, tumor size, molecular subtypes, the largest diameter decrease rate, and change of blood perfusion all played a significant role in predicting pCR, while molecular subtype was the strongest predictor. HER2-positive tumors ranked highest, with a pCR rate of more than half, whereas luminal A-like had a weak pCR rate, which was consistent with a previous large cohort study with a pCR rate only of only 0.3% in luminal A-like subtype (9, 32). Because of the utility of defining molecular subtype, our study reported a lower pCR rate of luminal A-like than previous studies. Luminal-like diseases, with inertness and favorable prognosis, represent strong endocrine sensitivity but poor chemosensitivity, especially luminal A-like diseases. Hormone receptor positivity is proven to be the weakest response to neoadjuvant treatment, with a significantly lower rate of pCR compared to negative patients (33). Although triple-negative was shown to have poor long-term survival with its aggressive characteristics, there was a significantly increased likelihood of achieving pCR, which could reliably differentiate individuals with excellent prognosis from those non-pCR but still had subtype-related invasion (9, 28, 34). With the advancement of neoadjuvant chemotherapy, optimizing the pCR rate is one of the objectives for triple-negative cancer, consequently helping to improve and enhance long-term survival.

Besides, time interval, tumor size and the change of blood perfusion measured by ultrasound were additional independent predictors of pCR. Since prolonged time interval was shown to be strongly associated with advanced stages, our study also demonstrated that short time interval might increase the possibility of pCR after adjusting for multivariable (35). It is beneficial for patients to undergo early diagnosis, approximately within the first 3 months after the emergence of symptoms, before worsening of the disease and tumor size. Patients with large tumor size showed significantly lower chances of achieving pCR compared with cT1 stage, which is in sync with recent studies (28, 36). Many studies have observed that tumor blood flow was shown to reduce significantly following NAC using ultrasound imaging, which was corroborated as a parameter to reflect response (37). Wan et al. reported predictive ability of change in blood perfusion by quantifying several related parameters before and after four cycles (38). Although we employed color Doppler ultrasound with only rough detection in the analysis, it was showed that reduction of blood perfusion was significantly correlated with pCR. Unfortunately, neither age or menopausal status did not have any significance in achieving pCR, which was consistent with a previous report (28). In addition, AR status failed to predict the clinical outcome of pCR, which might exert its effect in therapeutic target and drug resistance (39).

## Limitations

There were some limitations to our research. As a result of the study design, the data obtained retrospectively gave rise to introducing confounding factors and demonstrating inherent bias in this research. In addition, although our enrolled cohort consisted of 480 cases, no patient was found to achieve the pCR in the luminal A-like subtype, whose characteristics are associated with a low pCR rate. And the same condition happened with HER2 positive to predict non-responder. Therefore, accuracy, specificity, and sensitivity were limited to confirmation. Further studies with larger sample size are required to provide more better data and precise recommendations for tailored treatment.

## Conclusion

Our study calculated optimal cut-off points of the largest diameter decrease for predicting pathological response to NAC in patients with IBC among different molecular subtypes. The aggressive subtypes possessed higher pCR rates and the most accurate measurement by ultrasound. Besides, five independent predictors were identified where molecular subtypes played a decisive role. As a result, the utility of the developed nomogram is of clinical relevance, allowing patients with high aggressive to obtain greater benefit and take full advantage of few opportunities for prolonged survival.

## Data availability statement

The datasets presented in this article are not readily available because of protection of patient privacy. The datasets are accessible with reasonable request from the corresponding author. Requests to access the datasets should be directed to JG, gejie1980@tmu.edu.cn.

## Ethics statement

All procedures performed in this study which involved human participants were in accordance with the ethical standards of institutional and/or national research committees and with the 1964 Declaration of Helsinki and its later amendments or comparable ethical standards. This retrospective study was approved by the Institutional Review Board of Tianjin Medical University (Number: bc2022211).

## Author contributions

K-NW, Y-JM, YY and W-RC conceived this study. K-NW and Y-JM wrote the manuscript with input from all authors. JG, X-CC and XW took the lead and supervised the quality of this study. All authors contributed to the article and approved the submitted version.
