# Supplementary material for: Predicting pathological complete response after neoadjuvant chemotherapy: A nomogram combining clinical features and ultrasound semantics in patients with invasive breast cancer
Source: Front Oncol. 2023 Mar 22;13:1117538. doi: 10.3389/fonc.2023.1117538 (PMC10075137; doi:10.3389/fonc.2023.1117538)
Supplement: Supplementary file 1 [file DataSheet_1.docx]

**Predicting pathological complete response after neoadjuvant chemotherapy: a nomogram combining clinical features and ultrasound semantics in patients with invasive breast cancer**

Ke-Nie Wang^1, 2, 3, 4*^, Ya-Jiao Meng^5*^, Yue Yu^1, 2, 3, 4^, Wen-Run Cai^1, 2, 3, 4^, Xin Wang^1, 2, 3, 4^, Xu-Chen Cao^1, 2, 3, 4^, Jie Ge^1, 2, 3, 4#^

^1^the First Department of Breast Cancer, Tianjin Medical University Cancer Institute and Hospital, National Clinical Research Center for Cancer, Tianjin 300060, China.

^2^Key Laboratory of Cancer Prevention and Therapy, Tianjin 300060, China.

^3^Tianjin’s Clinical Research Center for Cancer, Tianjin 300060, China.

^4^Key Laboratory of Breast Cancer Prevention and Therapy, Tianjin Medical University, Ministry of Education, Tianjin 300060, China.

^5^Department of Obstetrics & Gynecology , Tianjin 4th Centre Hospital, Tianjin 300060, China.

^*^These authors contributed equally to this work.

**^#^Corresponding author:** Prof. Jie Ge, the First Department of Breast Cancer, Tianjin Medical University Cancer Institute and Hospital, Huan-Hu-Xi Road, He-Xi District, Tianjin 300060, China. Tel: 86-022-23340123 ext. 2074, E-mail: gejie1980@tmu.edu.cn


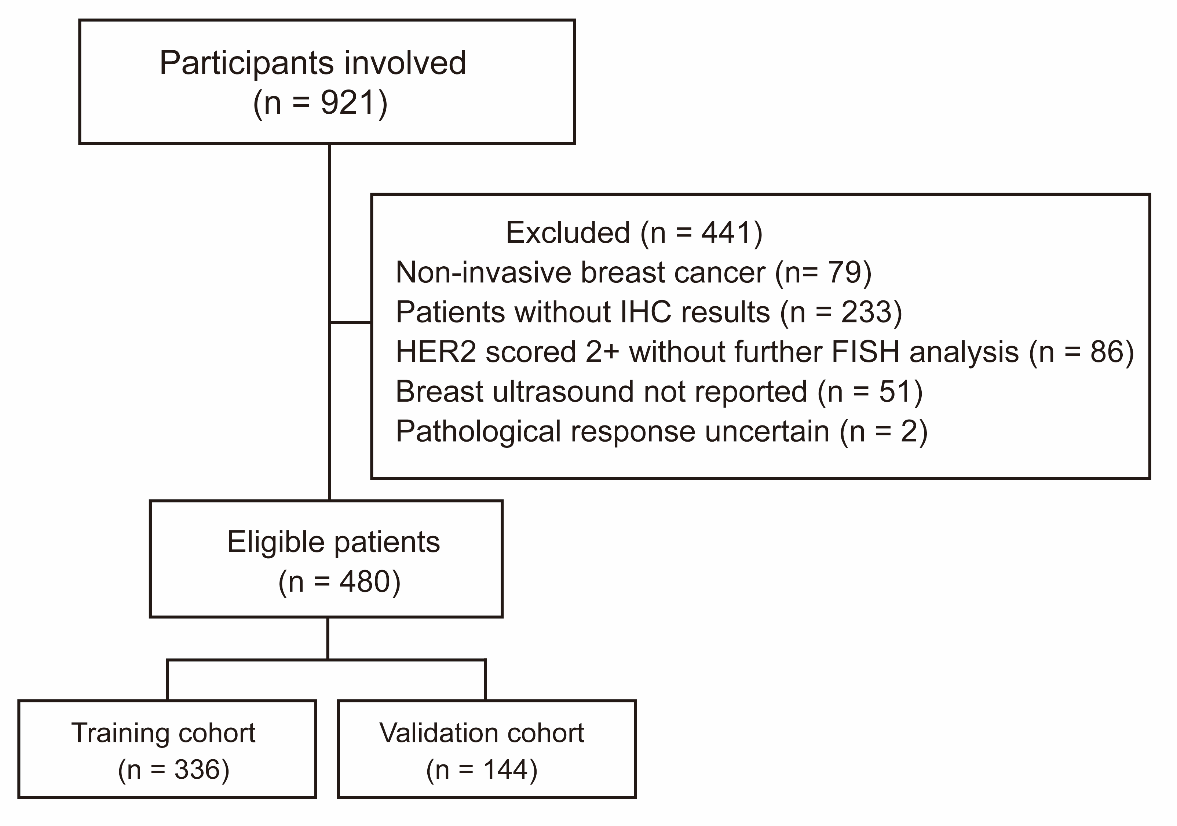


**Supplementary Figure 1.** Flow chart of the study design.

| **Molecular subtypes** | **All Patients**  **(n=480)** | **Training cohort（n=336）** | **Validation cohort (n=144)** |
| --- | --- | --- | --- |
| Luminal A-like | 0 (0.0%) | 0 (0.0%) | 0 (0.0%) |
| Luminal B-like (HER2 negative) | 33 (12.5%) | 19 (10.5%) | 14 (16.9%) |
| Luminal B-like (HER2 positive) | 19 (30.2%) | 13 (30.2%) | 6 (30.0%) |
| HER2 positive (non-luminal) | 19 (51.4%) | 15 (51.7%) | 4 (50.0%) |
| Triple-negative | 28 (39.4%) | 20 (37.7%) | 8 (44.4%) |
| Total | 99 (20.6%) | 67 (19.9%) | 32 (22.2%) |

**Supplementary Table 1.** Patients achieved pathological complete response following neoadjuvant chemotherapy in different molecular subtypes.

| **Molecular subtypes** | **pCR (pathologic response grade 3)** | | | **Responder (pathologic response grade 2)** | | | **Non-responder (pathologic response grade 1 and 0)** | | | |
| --- | --- | --- | --- | --- | --- | --- | --- | --- | --- | --- |
|  | **Cut-off**  **（%）** | **AUC** | **95% CIs** | **Cut-off**  **（%）** | **AUC** | **95% CIs** | **Cut-off**  **（%）** | **AUC** | | **95%** **CIs** |
| Luminal A-like | - | - | - | 21.62% | 0.779 | 0.527-1.000 | 18.83% | | 0.815 | 0.601-1.000 |
| Luminal B-like (HER2 negative) | 53.23% | 0.580 | 0.408-0.753 | 45.55% | 0.641 | 0.555-0.726 | 23.21% | | 0.613 | 0.430-0.796 |
| Luminal B-like (HER2 positive) | 51.56% | 0.668 | 0.457-0.879 | 16.11% | 0.709 | 0.527-0.891 | 15.50% | | 0.867 | 0.743-0.990 |
| HER2 positive (non-luminal) | 41.89% | 0.669 | 0.457-0.881 | 40.69% | 0.684 | 0.465-0.904 | 0.00% | | 0.929 | NA |
| Triple-negative | 53.52% | 0.896 | 0.790-1.000 | 53.16% | 0.830 | 0.723-0.938 | 16.51% | | 0.952 | 0.885-1.000 |
| Total | 53.26% | 0.696 | 0.614-0.778 | 44.75% | 0.697 | 0.640-0.753 | 23.21% | | 0.765 | 0.656-0.874 |

**Supplementary Table 2.** The optimal cut-off points of the largest diameter decrease for predicting different pathological response.

|  | Luminal A-like | Luminal B-like (HER2 negative) | Luminal B-like (HER2 positive) | HER2 positive (non-luminal) | Triple-negative | Total |
| --- | --- | --- | --- | --- | --- | --- |
| Accuracy | - | 0.762 | 0.721 | 0.724 | 0.925 | 0.774 |
| Sensitivity | - | 0.474 | 0.615 | 0.667 | 0.850 | 0.582 |
| Specificity | - | 0.796 | 0.767 | 0.786 | 0.970 | 0.822 |
| DOR | - | 3.518 | 5.257 | 7.333 | 181.333 | 6.413 |
| FPR | - | 0.204 | 0.233 | 0.214 | 0.030 | 0.178 |
| PPV | - | 0.214 | 0.533 | 0.769 | 0.944 | 0.448 |
| NPV | - | 0.928 | 0.821 | 0.688 | 0.914 | 0.888 |
| Youden index | - | 0.270 | 0.382 | 0.452 | 0.820 | 0.404 |

**Supplementary Table 3.** Breast ultrasound performance for predicting pathological complete response among molecular subtypes after NAC.

|  | Luminal A-like | Luminal B-like (HER2 negative) | Luminal B-like (HER2 positive) | HER2 positive (non-luminal) | Triple-negative | Total |
| --- | --- | --- | --- | --- | --- | --- |
| Accuracy | 0.600 | 0.669 | 0.814 | 0.724 | 0.736 | 0.679 |
| Sensitivity | 1.000 | 0.514 | 0.900 | 0.667 | 0.576 | 0.554 |
| Specificity | 0.538 | 0.771 | 0.615 | 0.818 | 1.000 | 0.788 |
| DOR | - | 3.552 | 14.400 | 9.000 | - | 4.612 |
| FPR | 0.462 | 0.229 | 0.385 | 0.182 | 0.000 | 0.212 |
| PPV | 0.250 | 0.597 | 0.844 | 0.857 | 1.000 | 0.696 |
| NPV | 1.000 | 0.706 | 0.727 | 0.600 | 0.588 | 0.668 |
| Youden index | 0.538 | 0.285 | 0.515 | 0.485 | 0.576 | 0.342 |

**Supplementary Table 4.** Breast ultrasound performance for predicting patients achieving grade 2 among molecular subtypes after NAC.

|  | Luminal A-like | Luminal B-like (HER2 negative)30% | Luminal B-like (HER2 positive)16 | HER2 positive (non-luminal)40 | Triple-negative17 | Total(Cut-off=20%) |
| --- | --- | --- | --- | --- | --- | --- |
| Accuracy | 0.980 | 1.000 | 1.000 | 1.000 | 1.000 | 0.980 |
| Sensitivity | 1.000 | 1.000 | 1.000 | 1.000 | 1.000 | 1.000 |
| Specificity | 0.773 | 1.000 | 1.000 | 1.000 | 1.000 | 0.773 |
| DOR | - | - | - | - | - | - |
| FPR | 0.227 | 0.000 | 0.000 | 0.000 | 0.000 | 0.227 |
| PPV | 0.979 | 1.000 | 1.000 | 1.000 | 1.000 | 0.979 |
| NPV | 1.000 | 1.000 | 1.000 | 1.000 | 1.000 | 1.000 |
| Youden index | 0.773 | 1.000 | 1.000 | 1.000 | 1.000 | 0.773 |

**Supplementary Table 5.** Breast ultrasound performance for predicting patients achieving grade 1 among molecular subtypes after NAC.

| **Variables** | **Nomogram** |
| --- | --- |
| **Time interval, months** |  |
| <= 3 | 1.2 |
| > 3 | 0 |
| **Tumor size, cm** |  |
| <= 2 | 17 |
| > 2 and <= 5 | 0 |
| > 5 | 3 |
| **Molecular subtypes** |  |
| Luminal A-like | 0 |
| Luminal B-like (HER2 negative) | 75 |
| Luminal B-like (HER2 positive) | 88 |
| HER2 positive (non-luminal) | 100 |
| Triple-negative | 92 |
| **The largest diameter decrease rate** |  |
| <= 53% | 0 |
| > 53% | 19 |
| **Change of blood perfusion** |  |
| Stable | 0 |
| Less | 9 |
| More | 1 |

**Supplementary Table 6.** The detailed scores of categories of each independent predictor in the nomogram.
